# The New Income Tax Rules

**Published:** 1920-05-01

**Authors:** 


### THE NEW INCOME TAX RULES.

From the official tables which have been published it will no doubt have been already seen by those interested that up to professional incomes of about £2,000 there is little to fear from the new tax rules, and in many cases something to be gained. The new rules are supposed to hit bachelors somewhat severely, but that does not much trouble the medical profession. The fact, however, is that there is no special scale provided for unmarried persons, except in the sense that applies also to widowers. Taking "unmarried" in that wide significance, it is the case that there is in some instances a considerable increase. Thus, assuming that there is no allowance obtainable for young children or for a housekeeper, and dismissing the matter of life assurance, an income of £700 will pay £114 instead of £94; £900 will pay £169 instead of £135; £1,000 will pay £196 instead of £150; £1,500 will pay £331 instead of £281; but the present tax of £450 on £2,000 will be raised to only £466.

When we turn to married couples, even though without young children (or rather without children who count for the children abatement), we find a slightly lessened tax on professional incomes up to £700; practically no change at £800 and £900; about £20 of an increase at £1,000 and £1,500; and actually £11 off at £2,000.

When the married couple have three children counting for abatement, the result is still better; thus at £2,000 there is a saving of £38.

We shall deal as briefly as possible with the principal new rules :—

*Basis of Assessment.*—At present this is the actual year's income for public appointments, and the average of the three preceding years for general practice. The former is to remain, but the latter is to be charged to the measure of the previous year instead of any average. It is not yet certain whether this change will take effect for the assessments in the new current year 1920-21, or will be postponed till one year later; probably the latter. When it does take effect, some relief will be given in cases where the transition from the one system to the others involves special hardship. This may require close watching.

*Earned and Unearned Income.*—The latter is now to be known as "investment" income. Relief to earned income is to be given in a totally new way. There are not to be different rates of tax for the two classes of income, but in the case of earned income, not the whole, but only nine-tenths are to be brought into account. Thus if a doctor makes £1,000 clear, and his wife and he between them have £400 from investments, the gross would be £1,300, not £1,400. This arises from knocking off 10 per cent. of the professional profit. Thus under the previous rules the discrimination in favour of earned income ceased when the total income from all sources exceeded £2,500, but now it is given absolutely without any income limit; only the sum to be knocked off is never to exceed £200. Thus professional incomes of £2,000 and £4,000 each shed a flat sum of £200, so that the one counts as £1,800 and the other as £3,800. This relief is not extended to super-tax.

*Exemption Limits.*—These are to be £150 for unmarried persons and £250 for a married couple. Thus a married man with a wife, five children counting for abatement, and one dependent relative, and paying £30 of a life premium, will be wholly exempt from income-tax if his income is professional only and does not exceed £470. This is by reason of the various allowances which are dealt with below.

*Personal and Marriage Allowance.*—An unmarried person, in the sense explained above, gets a free abatement of £135, and a man with a wife gets £225 free. There is no income limit.

*Wife Allowance.*—This now disappears as a separate head of claim, being merged in the marriage allowance just mentioned.

*Children Allowance.*—This is now £36 for a first or only child, and £27 for each other child. These are slightly increased figures, and there is now no limit of income; but if the child has a separate income equal to the allowance, there is no claim. In other respects the conditions remain as last year; general age restriction to sixteen, but no age restriction in the case of whole-time students.

*Dependent Relatives.*—£25 for each, as before, and now no income limit. Where the relative is the taxpayer's mother, she need be neither aged nor infirm.

*Housekeeper.*—The amount is £50, and it is available only to a widower or widow who pays a female relative to look after young children, or to an unmarried person who maintains a widowed mother or other female relative to look after young brothers or sisters. There is now to be no income limit.

*Graduation.*—Under the previous arrangements there was a complicated scale of rates of tax graduated to suit different incomes. All these go, but much the same result is brought out in a totally different way. You take the gross income. Then from that you take off all the various allowances which we have mentioned so far as applicable. What remains over is all to be taxed. But not all at the same rate, and that is the admission of the idea of graduation. The first £225 is taxed at *one-half* of the nominal or standard rate, and the balance, if any, at the full rate. This relief is given no matter how large the total income is, but, of course, the smaller the income the greater the proportion of the whole is the £225. Indeed, it may be the whole. We give an example below, but we may say at once that in the case we have already figured of a doctor with a wife, five children, one dependant, and a £30 life premium, he would pay no tax at a higher rate, at present, than 3s. per £ (being one-half of the present standard 6s. rate), unless his total income exceeded £720.

*Depreciation.*—Doctors are now to be allowed, if they prefer, to charge depreciation of plant and machinery, instead of renewals. Of course, they cannot get both. This applies to cars, and in some cases to great expensive technical appliances. In all cases repairs may be charged, and, of course, all running costs.

*Non-Cash Income.*—What we refer to here are such valuable assets as free residence, board, service, fuel, light, &c., attached to professional appointments. These are perhaps more common in the medical than in any other profession. Up till now they have not been regarded as taxable income. Now that is to be altered, and they will have to be included at their fair value. Whether this will be operative this year or not we do not yet know. In any case, we assume that it will be only the value to the particular medical man. Cases do actually occur when compulsory residence in a certain house is not worth anything like the ordinary annual value of the house, or may even be a minus quantity.

(*Continued on page* 126.)

### The New Income Tax Rules—(*continued from page* 115).

*Professional Dwelling-house.*—In the case of a house used both for practice and residence, two-thirds of the rent or annual value is not necessarily to be the limit of what may be deducted as expenses of the practice. The like will apply to rates. We never have admitted that there was any such limit in the case of house expenses, such as service, &c.

*Cash or Earnings.*—We are not aware that it is intended to withdraw the option which, at present, members of the profession enjoy of making up their accounts on either the cash or the earnings basis as they may prefer. In point of fact, the great majority adopt the cash basis, that is, ignoring all fees earned but not yet received, on the one hand, and all expenses incurred but not yet paid, on the other hand.

*Life Assurance.*—The conditions remain as before, but the manner of giving the relief is quite new. As regards policies effected after June 1916, the allowance is to be at one-half of the standard rate of tax. As regards earlier policies the allowance is to be at one-half of the standard in the case of incomes not exceeding £1,000, at three-quarters of the standard for incomes not exceeding £2,000, and at the full standard rate on higher incomes.

*Casual Income.*—It appears probable that Stock Exchange gains, and other things which have previously passed as capital increments, will now be roped in as taxable income. But we have not space to do more than barely mention this.

*Super-tax.*—This is now to apply absolutely to all incomes exceeding £2,000 so far as regards the excess. Up till now it has not applied at all unless the income exceeded £2,500, though then it did apply back to £2,000. And these rates are a good deal stiffened, as the following examples prove :—

| Incomes over £2,000 | Old Rate s. d. | New Rate s. d. |
|---|---|---|
| £500 up to £2,500 (when total not over £2,500) ... ... | Nil | 1 6 |
| £500 up to £2,500 (when total over £2,500) ... ... ... ... | 1 0 | 1 6 |
| Next £500 up to £3,000 ... | 1 6 | 2 0 |
| Next £1,000 up to £4,000 ... | 2 0 | 2 6 |
| Next £1,000 up to £5,000 ... | 2 6 | 3 0 |

Now if the allowances apply in the case of super-tax, not even life premiums, the tax is charged on the previous year's income as adjusted for income-tax, and without deducting the income-tax. Many people consider it a great grievance that super-tax is thus, in a sense, charged on income-tax.

*Example.*—It must suffice to give one example :—

| | | |
|---|---|---|
| Net professional income after making all proper deductions from gross drawings. This income may be on the three years' average, or it may be last year's income; that depends on the final demand of the Chancellor of the Exchequer | | £1,500 |
| Wife's investment income ... ... ... ... ... | | 700 |
| Own investment income ... ... ... ... ... | | 200 |
| | | £2,400 |
| Allow off :— | | |
| 1. One-tenth of the professional income | £150 | |
| 2. Marriage allowance ... ... ... ... | 225 | |
| 3. Two children over sixteen—whole-time profits or students, £36 and £27 | 63 | |
| 4. Three children under sixteen—£27 each ... ... ... ... ... ... | 81 | |
| 5. One dependent relative ... ... ... ... | 25 | |
| 6. Life premiums, on pre-1916 policies, £600, but this cannot all be allowed, for it exceeds one-sixth of the total income; therefore restrict to one-sixth of £2,400, which is ... ... ... | 400 | |
| | | 944 |
| Taxable income ... ... ... ... | | £1,456 |

This £1,456 is all taxable, but at what rate or rates? The answer is that £225 is chargeable at 3s. and £1,231 at 6s. This gives a total tax payment of £403. On the same figures and facts the tax last year would have been £535. But now there will be liability for £30 of super-tax, while previously there would not. The reason of the great relief in such a case as this is the number of children eligible for the children allowance, and the fact that the family allowances are now to be given without any income limit.

